# Gouty Arthritis Treatment: Advancements in Topical Lipid-Based Nanocarrier Delivery Systems

**DOI:** 10.34172/apb.44012

**Published:** 2025-04-04

**Authors:** Shubham Kumar, Shreya Kaul, Neha Jain, Chirag Jain, Manisha Pandey

**Affiliations:** ^1^Centre for Pharmaceutics, Amity Institute of Pharmacy, Amity University, Noida, India.; ^2^Department of Industrial Pharmacy, Amity Institute of Pharmacy, Amity University, Noida, India.; ^3^Department of Pharmaceutical Sciences, Central University of Haryana, Mahendergarh, 123031, India.

**Keywords:** Liposomes, Nanostructured lipid carriers (NLC), Nanoemulsions, Transferosomes, Gout

## Abstract

The formation of urate crystals in the joints causes severe, erratic flare-ups of joint pain, swelling, and erythema in gout, one kind of inflammatory arthritis. The standard treatment currently available involves the use of nonsteroidal anti-inflammatory drugs (NSAIDs), colchicine, allopurinol, febuxostat, and corticosteroids which require lifelong management via oral or parenteral route. The challenge is the therapy adherence as the symptoms become better, patients may quit taking them, which could result in more episodes. In addition, conventional therapy regimes demonstrate insufficient effectiveness and minimal safety owing to these drug molecule’s biopharmaceutical limitations, including inadequate chemical stability and an insufficient capacity to target the pathophysiological pathways. Therefore, developing an alternative drug carrier system that can meet the challenge is necessary. In recent years, the use of lipid-based nanocarriers has increased due to their properties of enhancing solubility and bioavailability of poor-soluble drugs, site-specific targeting, and sustained release. In this review, an attempt has been made to highlight the challenges of available therapies for gout along with its pathophysiology, the mechanism of lipoidal nanocarriers permeation via topical route, and recent advancements in gout therapy using lipid nanocarriers based on preclinical experiments. In addition, patents and clinical trials of lipid-based nanocarriers have also been discussed. Lipid-based nanocarriers present a potential strategy specifically for topical gout therapy as this can offer localized therapy with minimal systemic exposure. Even though lipid-based nanocarriers show promise for gout topical therapy, several issues that need to be looked after, including economically viable scalability and regulatory approvals.

## Introduction

 Gouty arthritis (GA), clinically referred to as gout, is a type of crystalline arthropathy marked by recurrent, acute episodes of intense pain, erythema, and edema in the affected joints. This condition predominantly impacts the articulations of the first metatarsophalangeal joint and other articulations, including the ankles, knees, elbows, wrists, and small joints of the hands.^1^ Current and forecasted gout epidemiology data of future mortality were obtained from the Global Health Data Exchange (GHDx) registry and the World Health Organization (WHO) database. Gout incidence, prevalence, and health loss have all grown significantly over the last 25 years, and they are all greater in men than in women.^2^ In 2020, 55.8 million (95% CI 44.4-69.8) persons worldwide had gout, with an age-standardized prevalence of 659.3 (525.4-682.3) per 100 000, a 22.5% rise (20.9-24.2) since 1990. In 2020, the global frequency of gout was 3·26 (3·11-3·39) times greater in males than in females, and it rose with age. In 2050, the total number of gout cases is expected to reach 95·8 million (81·1-116), with population growth accounting for the majority of the increase and the anticipated change in gout prevalence making just a tiny contribution. In 2050, the age-standardised gout prevalence is expected to be 667 (531-830) per 100 000 people. In 2020, the global age-standardised YLD rate of gout was 20·5 (14·4-28·2) per 100,000 people. According to the projection model, the number of people with gout will rise by more than 70% between 2020 and 2050, owing mostly to population growth and aging. Gout prevalence rises with age and correlates with the socio-demographic index (SDI). High SDI locations have a > 3-fold greater incidence risk of gout compared to low SDI regions. Given the link between gout handicap and high BMI, population-wide dietary and lifestyle changes aimed at reducing body weight are required, as well as access to therapies to prevent and control flares.^3^

 The pathophysiology of gout is rooted in hyperuricemia, a state of elevated serum uric acid (UA) levels resulting from the metabolism of purines. UA, a breakdown product of purine nucleotides, normally dissolves in the blood and is excreted via the kidneys.^2^ Monosodium urate (MSU) crystals precipitate in the synovial fluid and other tissues because of supersaturation brought on by either overproduction or underexcretion of UA, which is the cause of hyperuricemia. By activating the NLRP3 inflammasome, these crystals cause the production of pro-inflammatory cytokines such as interleukin (IL)-1β, IL-8, and tumor necrosis factor (TNF-α), which recruits neutrophils and other immune cells to the site of deposition and causes a strong inflammatory response. These UA crystals can also form deposits known as tophi and contribute to kidney stones.^3,4^ Chronic GA is characterized by the presence of tophi, which are lumps of urate crystals accumulated in and around the joints. When body fluids have UA concentrations higher than 0.42 mmol/L, they become supersaturated, which causes these crystals to form and subsequently cause gout and accompanying symptoms. These tophi can cause joint destruction and deformity, contributing to disability and reducing the quality of life for those affected.^5,6^

 The treatment of GA aims to relieve acute symptoms and prevent future attacks, primarily by managing UA levels and inflammation. The management of GA involves several conventional formulations, including oral, parenteral, topical, and monoclonal antibodies.^7^ Pain and inflammation are frequently reduced by oral formulations, such as nonsteroidal anti-inflammatory drugs (NSAIDs) like ibuprofen and naproxen. Colchicine is a treatment option for gout, particularly when NSAIDs are unsuitable or ineffective.^8^ In more severe cases, corticosteroids such as prednisone may be used due to their strong anti-inflammatory properties. This approach is often considered when NSAIDs and colchicine prove ineffective or are contraindicated.^9^ Long-term management of GA involves urate-lowering therapies (ULTs) to maintain serum UA levels below the saturation point of MSU. ULTs such as allopurinol and febuxostat (xanthine oxidase inhibitors), directly inhibit the enzyme xanthine oxidase, which is responsible for producing UA from hypoxanthine.^10^ Probenecid, a uricosuric agent, increases UA excretion by inhibiting its reabsorption in the kidneys. Parenteral formulations, offer rapid relief and are typically used in acute settings, examples include corticosteroids such as methylprednisolone and triamcinolone, as well as intramuscular administration of NSAIDs like ketorolac.^11^ Pegloticase is reserved for severe cases and is administered in a clinical setting. Topical formulations provide localized anti-inflammatory effects and include options such as diclofenac gel, capsaicin cream, and hydrocortisone cream. Newer anti-inflammatory therapies includeIL-1 inhibitors (anakinra, canakinumab, gevokizumab, and rilonacept), bucillamine, caspase inhibitors (pralnacasan), NLRP3 inflammasome inhibitors, and recombinant AAT-Fc.^10^

 However, these traditional treatments face several challenges.The oral medications are convenient for patients but often cause gastrointestinal (GIT) side effects such as stomach pain, heartburn, bleeding, and ulcers.^12^ Long-term use can lead to kidney damage and an increased risk of cardiovascular events. Moreover, they may have inconsistent bioavailability.^13^ Parenteral formulations, despite their effectiveness and rapid action, are not suitable for chronic use due to the discomfort and risk of infections from repeated injections. As these are invasive and can be painful at the injection site.^14^ Topical formulations minimize systemic side effects but often suffer from limited skin penetration, reducing their efficacy in reaching deeper joint tissues where urate crystals are deposited.^15^ Moreover, topical formulations can cause local skin reactions such as rash, itching, dryness, burning, stinging, and redness at the application site. Their systemic absorption is low, but there is still a risk of GIT and cardiovascular side effects.^16^ Monoclonal antibodies represent a newer approach; however, these are associated with high costs and can induce immunogenicity, leading to reduced efficacy over time and potential allergic reactions.^17^ IL-1 Inhibitors may result in hypersensitivity reactions, injection site reactions, and an elevated risk of infections. Long-term use may also impact liver function and blood cell counts. These challenges underscore the need for innovative delivery systems to enhance the therapeutic outcomes of GA treatments while minimizing side effects.^18,19^

 In this regard, lipid-based nanocarrier systems have emerged as a promising solution. Compared to conventional formulations, nanocarriers such as cubosomes, transferosomes, niosomes, ethosomes, solid lipid nanoparticles (SLNs), nanostructured lipid carriers (NLCs), and liposomes have several advantages.^20^ Lipid nanoparticles (NPs) are a highly advantageous platform for the topical delivery of therapeutic agents compared to conventional approachesfor GA due to their unique properties and composition. One of the primary benefits of lipid NPs is their ability to enhance drug penetration through the skin. The lipids in these NPs closely resemble the natural lipids in the skin’s stratum corneum, which allows them to integrate seamlessly with the skin barrier, improving the delivery of encapsulated drugs to deeper layers.^21^ Additionally, the surface properties of nanocarriers can be modified to improve their interaction with biological membranes, further enhancing their penetration and retention in the target tissues.^22^ This targeted delivery approach is particularly beneficial for managing GAs, as it addresses the need for high local drug concentrations to dissolve urate crystals and alleviate inflammation effectively.^23^ The ability to regulate and maintain the release of encapsulated medications for a prolonged amount of time is provided by lipid nanocarriers. By maintaining therapeutic levels of the medication in the target tissues through a controlled release mechanism, application frequency can be decreased, and patient compliance can be increased.^24^ In GA, sustained release of anti-inflammatory agents from lipid nanocarriers can provide prolonged pain relief and reduce the need for frequent dosing, which is beneficial for managing chronic symptoms.^25^ Moreover, the targeted delivery capabilities of lipid-based nanocarriers can minimize off-target effects and reduce the risk of adverse side effects, addressing a major limitation of conventional treatments. This targeted approach not only enhances the treatment’s efficacy but also improves its safety profile, making it a viable option for patients with comorbid conditions or those intolerant to standard therapies.^26^

 This work intends to highlight the limits of traditional GA treatment strategies and study the developed alternative topical drug delivery methods specifically based on lipid-based nanocarriers. The keywords for the recent research review were limited to lipid nanoparticles, lipid nanocarriers, topical delivery, gout, and GA. The study focuses on the pathophysiology of gout, the mechanics of lipid-based nanocarrier penetration via the skin, and advances in topical nanocarrier formulations that were found during the literature search such as transferosomes, NLCs, SLNs, ethosomes, cubosomes, nanoemulsions, and self-nanoemulsifying drug delivery systems (SNEDDS). The study also explores patents and clinical studies for lipid-based nanocarrier applications, highlighting their potential for targeted, patient-friendly, and efficient GA therapy.

###  Pathophysiological mechanisms in gouty arthritis: from uric acid to tophus formation

**Figure 1:**
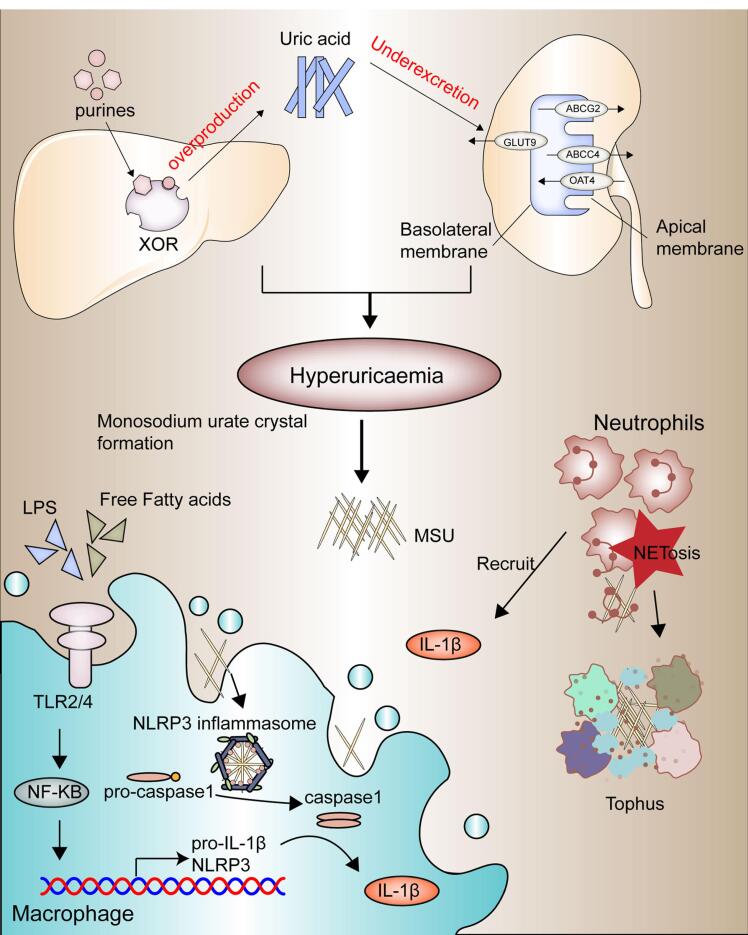
The pathophysiology of GA. progresses through four primary stages: the emergence of a hyperuricemic state, MSU crystal formation, and deposition, acute GA triggered by crystal deposition and inflammation, and chronic inflammation leading to bone erosion and tophi formation. Reprinted under licence CC BY 4.0.^27^

 The pathophysiology of GA (Figure 1) progresses through four primary stages: the emergence of a hyperuricemia state, MSU crystal formation and deposition, acute GA triggered by crystal deposition and inflammation, and chronic inflammation leading to bone erosion and tophi formation.^27^

###  Hyperuricemia 

 Hyperuricemia is a critical precursor to gout. It occurs when urate, the final metabolite produced by the metabolic pathway of purine nucleotides, accumulates in the blood. Urate functions as an antioxidant, an immune response signal, and a regulator of blood pressure in low-salt diets. Environmental factors, such as consumption of purine-rich foods (e.g., beer, meats, seafood), are linked with hyperuricemia and gout.^28^ Fructose consumption elevates the activity of xanthine oxidase, the enzyme responsible for the final step of purine nucleotide degradation,leading to higher serum urate levels. Alcohol also contributes to hyperuricemia through its metabolism into acetate, consuming ATP, and generating AMP. Conversely, dietary factors such as coffee, low-fat dairy, and vitamin C can lower urate levels.^29^ The rapid cell division in conditions such as psoriasis and myeloproliferative disorders leads to increased purine nucleotide breakdown, resulting in elevated serum urate levels due to excessive urate production.^30^ The kidneys and the gut act in concert to control urate excretion, and any deficiency in this process can result in hyperuricemia.Kidney excretion’s pivotal role is highlighted by the direct correlation between serum creatinine and urate levels.^31^ Conditions such as elevated insulin levels in obesity, renal disease, metabolic syndrome, and diuretic therapy (e.g., furosemide) are associated with UA accumulation triggering gout manifestation. Urate undergoes free filtration through the glomeruli in the kidney, and its elimination is subsequently regulated by proximal tubule urate transporters.^32^ Key transporters on the apical surface include OAT4 (SLC22A11), URAT1 (SLC22A12), GLUT9 (SLC2A9), and OAT10 (SLC22A13), which facilitate urate movement from the lumen into cells, while secretory transporters such as NPT1 (SLC17A1), ABCG2 and ABCC4 manage urate secretion. Basolateral transporters, including OAT1 (SLC22A6), OAT2 (SLC22A7), OAT3 (SLC22A8), and GLUT9, control urate reabsorption.^33,34^ The exact mechanisms of urate transport in the gut remain elusive but are likely to involve similar transporter proteins. Genetic variants in ABCG2, are crucial for understanding overall urate homeostasis, as genetic variants in this transporter can lead to decreased extrarenal urate excretion resulting in hyperuricemia.^35^ Genetic variations in genes encoding urate transporters, such as SLC2A9 (GLUT9), SLC22A12 (URAT1), and ABCG2, have been strongly linked to variations in serum urate levels and gout risk. These genetic factors often play a more significant role in hyperuricemia compared to dietary factors in modern environments.^36^

###  MSU crystal deposition

 MSU crystal formation marks the progression of gout. Approximately 25% of hyperuricemic individuals show crystal deposits detectable by imaging, particularly in the first metatarsophalangeal joint, midfoot, and Achilles tendon.^37^ MSU crystals, are visible under a microscope as negatively birefringent needle-shaped structures and are composed of stacked purine ring sheets at the molecular structure.^38^ The process of crystallization generally involves three stages: supersaturation, triggered by reduced solubility, which then promotes nucleation and subsequent crystal growth.^39^ High urate concentration is crucial for these stages. Laboratory analysis shows that MSU crystallization occurs at urate levels exceeding 0.41 mmol/l (6.8 mg/dl) at 37 °C and pH 7.0.^40^ Urate solubility decreases further in the pH 7–8 range and with elevated sodium ion levels. Factors such as lower temperatures, pH levels between 7-8, and elevated sodium ion levels reduce urate solubility, promoting crystal formation.^41^ Additional contributors to MSU crystallization have been identified, including connective tissue elements, bovine cartilage matrix components, synovial fluid components, and anti-urate antibodies.^42^

###  Acute inflammatory response to MSU crystals

 MSU crystals act as damage-associated molecules, triggering innate immune pathways. Activated NLRP3 inflammasomes in monocyte-macrophage lineage are particularly important in triggering gout flares. Activation requires a two-signal process: the first signal, often mediated by TLR4 and TLR2 stimulates NF-κB, leading to the generation of IL-1β precursors and inflammasome components.^43^ The second signal, provided by MSU crystals, activates caspase-1, which converts pro-IL-1β to its active form, IL-1β. IL-1β then binds to its receptor, initiating a cascade of pro-inflammation mediators that recruit neutrophils and other immune cells to the crystal accumulation area.^44^ The 2-step activation process of the NLRP3 inflammasome explains why the presence of MSU crystals alone doesn’t inevitably lead to an inflammatory response. Attributes that include fatty acid metabolites, gut microbiome, and microbial metabolites can trigger inflammation in the occurrence of MSU crystals.^45^ The metabolic signaling molecule AMPK (PRKAA2), known for its anti-inflammatory properties, suppresses the activation of the signaling cascades.^46^ Neutrophils, once recruited, attempt to phagocytose the MSU crystals but often undergo inflammatory death, releasing their contents into the extracellular space.^47^ This process includes the release of neutrophil extracellular traps, which immobilize and neutralize pro-inflammatory molecules, helping to resolve the gout flare. Other anti-inflammatory factors like IL-1ra, IL-10, TGF-β, and IL-37 also contribute to inflammation resolution.^48^

###  Chronic inflammation and tophi formation

 Chronic GA results from repeated acute flares, leading to persistent inflammation and the formation of tophi. Tophi are aggregates of MSU crystals surrounded by a matrix of lipids, proteins, and mucopolysaccharides.^49^ They establish a microenvironment that incorporates adaptive and innate immune cells, MSU, and fibroblasts, which stimulate bone resorption by osteoclasts while inhibiting bone formation by osteoblasts, leading to bone erosion.^50^ The prolonged inflammatory response driven by elevated pro-inflammatory cytokines, particularly IL-1β, plays a significant role in bone and cartilage damage.^51^ IL-1β is essential for neutrophil recruitment and the perpetuation of inflammatory processes. Activation of the IL-1 receptor on endothelial cells is crucial for the transcription of other pro-inflammatory cytokines and chemokines, exacerbating the inflammatory cycle.^52^

## Currently available treatment for gouty arthritis

 Conventional drugs for treating GA include NSAIDs, anti-gout agents, uricosuric agents, uricase enzyme inhibitors, xanthine oxidase inhibitors, corticosteroids, and URAT1 inhibitors as depicted in Table 1.^7^ NSAIDs like ibuprofen and naproxen are used to alleviate pain and inflammation but can cause GIT issues and cardiovascular problems with prolonged use.^53^ Xanthine oxidase inhibitors, like allopurinol and febuxostat, lower UA production but may cause rash, liver toxicity, and renal impairment.^54^ Anti-gout agents such as colchicine help reduce inflammation during acute flares but may lead to GIT side effects such as diarrhea and nausea.^55^

**Table 1:** Comparison of drugs used for gout management

**Category**	**Drug**	**Pharmacodynamic**	**Marketed drug**	**Averse effect**	**Reference**
NSAIDs	Naproxen	Inhibit cyclooxygenase (COX)-1 and COX-2, leading to decreased synthesis of prostaglandins	Aflaxen, Anaprox, Naprelan, Naprosyn (Tablet)	GIT issues such as stomach pain, GIT bleeding, heartburn, nausea and an increased risk of ulcers.	^56^
	Ibuprofen		Brufen (Tablet), Fenfid (Gel), Caldolor (Injection)	GIT issues such as stomach pain, heartburn, nausea, increased risk of ulcers and GIT bleeding. Prolonged use increases cardiovascular problems.	^53^
	Diclofenac	Inhibits both COX-1 and COX-2 enzymes, has a slightly higher affinity for COX-2. This inhibition reduces prostaglandin synthesis.	Reactin (Tablet), Diclolab (Gel), Voveran (Injection)	GIT irritation, ulcers, bleeding. Increased risk of cardiovascular issues. Prolonged use can cause liver toxicity.	^57^
	Allopurinol	Hinders xanthine oxidase, an enzyme within the purine breakdown pathway responsible for converting hypoxanthine into xanthine and eventually into UA.	Zyrick (Tablet), Zyloprim (Tablet), Logout – SR (Capsule)	Maculopapular pruritic rash, GIT adverse effects, liver necrosis, interstitial nephritis and hypersensitivity reactions such as Stevens-Johnson syndrome.	^58^
	Febuxostat	Febuxostat also inhibits xanthine oxidase but is more selective and potent compared to allopurinol.	Febucip adenuric Uloric, Febutaz (Tablet)	Liver function abnormalities, dizziness, nausea, rash, increased risk of cardiovascular events	^59^
Anti-gout agents	Colchicine	It interferes with cytoskeletal functions by hindering the formation of microtubules through the inhibition of beta-tubulin polymerization. Which disrupts the mobility of neutrophils, reducing their ability to migrate to inflamed areas and thus decreasing inflammation associated with gout attacks.	Colshine (Tablet), Zycolchin (Tablet), Mitigare (Capsule), Gloperba (Oral solution)	GIT-abdominal cramping and pain, sensorimotor neuropathy, myopathy, myalgia, alopecia, rash, azoospermia, oligospermia	^60^
Uricosuric agents	Probenecid	It inhibits URAT1 and various other anion transporters, leading to an enhanced renal excretion of UA through decreased reabsorption.	Bencid (Tablet), Benemid (Tablet)	UA urolithiasis, GIT intolerance, dizziness, headaches, rash, hemolytic anemia, sore gums, renal colic and exacerbation of gout.	^56^
Uricase enzyme inhibitor	Pegloticase	It works by converting UA into allantoin, a more soluble and easily excreted substance, through its enzymatic action as a recombinant uricase. This reduces UA levels and helps manage chronic gout.	Krystexxa (Injection), Piokind (Tablet), Pioglit (Tablet)	Infusion reactions, nausea, bruising, Constipation, gout flares, anaphylaxis, Nasopharyngitis, and hemolysis, particularly in patients with G6PD deficiency.	^61^
Corticosteroid	Prednisone	They work by binding to glucocorticoid receptors, altering gene expression, and inhibiting the release of substances that cause inflammation. They suppress the immune system by reducing the activity of lymphocytes and inhibiting the production of pro-inflammatory cytokines.	Omnacortil (Tablet), Wysolone (Tablet), Prednilead (Injection)	Increased appetite, weight gain, insomnia, mood changes, and elevated blood sugar levels, hypertension, osteoporosis, adrenal suppression, and cataracts.	^62^
	Methylprednisolone		Medrone (Tablet), Zempred (Tablet), Solu-Medrol (Injection), Advantan (Cream)	Facial rounding, puffiness, fat deposition, increased hair growth on the face, thighs, and trunk, insomnia, weight gain, elevated blood sugar, osteoporosis, acne, and increased appetite.	^63^
	Dexamethasone		Decadron (Injection), Dexasone (Tablet), Dexabliss (Tablet)	Insomnia, mood changes, increased blood sugar, weight gain, fluid retention, electrolyte imbalances, acne, nausea, digestive issues, adrenal insufficiency and osteoporosis,	^64^
URAT1 inhibitors	Lesinurad	Inhibits the function of URAT1 in the kidneys, which reduces the reabsorption of UA and increases its excretion in the urine, thereby lowering serum UA levels and helping manage gout.	Zurampic (Tablet)	Headache, upper respiratory tract infections, reflux esophagitis, increased blood creatinine levels, acute renal failure.	^65^

 Uricosuric agents, including probenecid, increase UA excretion but can result in kidney stones and GIT discomfort.^56^ Uricase enzyme inhibitors, such as pegloticase, break down UA into more soluble compounds but can cause allergic reactions and infusion-related side effects.^66^ Corticosteroids, such as prednisone, are effective in controlling severe inflammation but can lead to weight gain, osteoporosis, and increased infection risk with long-term use.^67^ Lastly, URAT1 inhibitors like lesinurad enhance UA excretion but can cause kidney function impairment and cardiovascular events. Managing these side effects is crucial for the safe and effective treatment of GA^68^.

## Lipid-based nanocarriers

**Figure 2:**
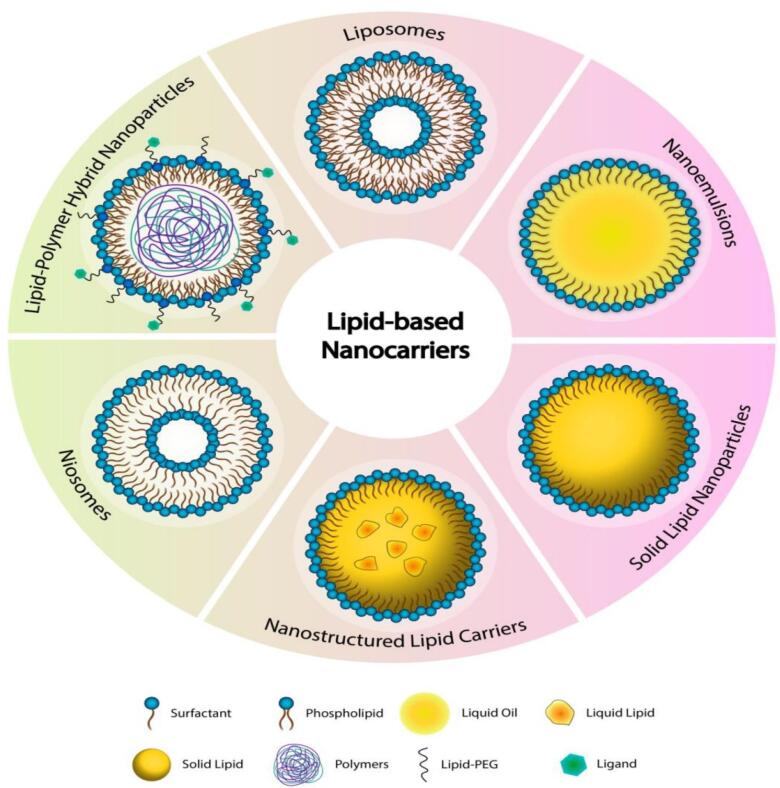
Structure of various types of lipid-based nanocarriers. Reprinted under licence CC BY 4.0^71^

 Lipid-based nanocarriers, such as SLNs, NLCs, and transferosomes, have emerged as promising drug delivery platforms due to their biocompatibility, ability to encapsulate both hydrophilic and lipophilic drugs, and targeted delivery potential. These nanocarriers are typically non-spherical in form, which is caused by an electrostatic contact between the polar/ionogenic phospholipid head and the solvent, or by non-polar lipid hydrocarbon moieties present in the solvent. The unique physicochemical qualities of LNs, which take the form of liposomes or solid core lipid nanoparticles with great biocompatibility, make them ideal carriers for medicines and food applications.^69^ These LNs, which are composed of homogeneous lipid bilayers or solid cores, can entrap a variety of cytotoxic medicines. The hydrophilic medication will be trapped in the water, but the lipophilic drug will be trapped in the lipid leaflets.^70^ The structures of various types of lipid nanocarriers have been depicted in Figure 2.

 SLNs are made up of a solid lipid matrix that remains solid at normal body temperature. SLN may be manufactured using several processes, including heat or cold homogenization, which is simple to scale up production, has high reproducibility, and does not use harmful organic solvents. Triglycerides, fatty acids, steroids, and biowaxes are commonly employed to construct SLN systems. Because of their tiny size and wide surface area, SLN is appropriate for coating with functionalized ligand moieties, antibodies, and other functional groups. They provide great physical stability, regulated drug release, and protection against degradation for sensitive pharmaceuticals, but they have disadvantages such as poor drug-loading capacity and probable drug ejection during storage.^72^ NLCs outperform typical lipid-based nanocarriers such as nanoemulsions, liposomes, and SLNs in terms of drug delivery due to their solid matrix at room temperature. They have higher physical stability, drug loading capacity, and biocompatibility.^73^ NLCs are made up of a combination of solid and liquid lipids, with regulated amounts for better bioactive retention and release characteristics. They are biocompatible systems with a stiff shape, which contributes to their distinctive features. NLCs are classified into three types: structurally distinct lipids, amorphous lipids, and solid-liquid mixtures.^74^ Common NLCs encapsulate better than solid lipids, but surface functionalization is problematic.^75^

 Transferosomes are ultra-deformable vesicular carriers that are specifically engineered for efficient medication administration via transdermal and topical methods. They are made up of a phospholipid bilayer mixed with edge activators such as surfactants or bile salts, which give their structure extraordinary flexibility. Transferosome’s deformability enables them to pass through small pores and intercellular gaps in the skin, allowing for deeper tissue penetration than normal liposomes.^76^ Transferosomes, which are typically between 100 and 300 nm in size, are highly biocompatible and may encapsulate both hydrophilic and lipophilic medicines. Their enzymatic degradation resistance and prolonged release features improve medicinal effectiveness. Transferosomes are physiologically stable, making them suited for a wide range of applications, such as peptide, hormone, and anti-inflammatory drug administration. Their versatility has made them an exciting tool in dermatology and other scientific domains.^77^

 SLNs are commonly used for regulated drug release and targeting, making them excellent for delivering anti-inflammatory medicines, anticancer medications, and antioxidants. Their use in gout treatment has shown potential by administering NSAIDs such as indomethacin while providing prolonged release and decreasing GIT adverse effects. NLCs, with their mixed solid and liquid lipid matrix, increase medication loading capacity and are beneficial in the treatment of chronic ailments such as dermatological disorders and systemic disease.^78,79^ They are also being investigated for gout therapy by administering UA-lowering medications such as allopurinol or febuxostat, which ensures longer drug activity and better patient compliance. Transferosomes, recognized for their exceptional skin penetration, are ideal for the transdermal administration of peptides, hormones, and anti-inflammatory medications.^76^ In gout, they can apply colchicine or NSAIDs topically, avoiding systemic adverse effects and treating inflamed joints specifically. These lipid carriers provide diverse alternatives for improving medication administration in gout and other therapeutic areas, such as cancer, skin diseases, and pain management.^23,80^ Top of Form

## Mechanism of lipid-based nanocarrier penetration

**Figure 3:**
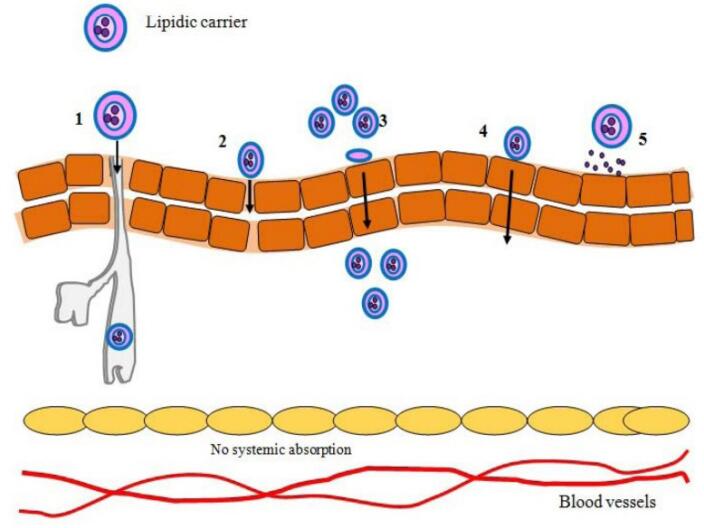
Various mechanisms of lipid-based nanocarriers topical skin cell penetration 1) pilosebaceous-mediated delivery, 2) intercellular transport, 3) skin lipid integration, 4) transcellular transport, and 5) absorptive transport.^70^

 Lipoidal nanoparticles possess the ability to improve the diffusion of therapeutic moieties across the skin by their nano-size and closer association with the epidermis. The process of medication delivery from lipoidal nanoparticles has been extensively studied, even though it is still a highly challenged subject. Lipid-based nanocarriers have developed as new medication delivery technologies that use their unique structural features to penetrate the stratum corneum barrier. The mechanisms of skin penetration for different lipid-based nanocarriers have been depicted in Figure 3. The skin’s outermost layer is mostly made up of corneocytes embedded in a lipid matrix that acts as a vital barrier against penetration. There are four basic avenues for lipid-based nanocarriers to permeate the skin. The first process is the unbroken passage of drug-laden vesicles into the epidermal layers. Depending on size and composition, these vesicles can pass through the stratum corneum, sending their contents further into the epidermis and dermis. The second process uses lipid vesicles to increase penetration. These vesicles improve drug diffusion through the epidermal barrier by fluidifying the lipid domains inside the stratum corneum. The third mechanism involves a direct interchange of carrier and skin lipids, known as “collision complex transfer.” Drugs intercalated in lipid bilayers can move to the stratum corneum’s surface phase, allowing for deeper penetration. Finally, lipid vesicles can improve transdermal distribution through appendageal routes such as hair follicles and sweat ducts. This channel provides new routes of drug entry beyond the usual transepidermal pathways.^23^

 Lipid nanoparticle penetration through the skin is controlled by several factors, including adherence to the skin, occlusive, hydration, diffusion, electrovalent bond formation, and thermodynamics, amongst others.^81,82^ The stratum corneum, the uppermost layer of skin, is mainly composed of dead cells called corneocytes that are surrounded by a lipid matrix. Since these bilayers of fatty acids and lipid-based nanocarriers have comparable lipid compositions, they can combine or disrupt each other, allowing the nanocarrier or its loaded drug to diffuse via the skin’s barrier. Gupta et al formulated lipid nanoparticles of three different actives i.e., tretinoin, clotrimazole, and ferulic acid using five types of lipids viz. Gelucire, Geleol, Compritol, Precirol, and Gelot, and evaluated them for penetration with the help of molecular dynamics simulations. The more powerful drug-skin association than the drug-nanoparticle association acts as the impetus for the transfer, which starts during the penetration stage.^83^ Deeper penetration can be facilitated by certain nanocarriers’ ability to squeeze and fit inside the epidermal narrow intercellular gaps. Malviya et al formulated sinapic acid lipid vesicle-based hydrogels and evaluated them for skin permeation. The outcomes revealed that the vesicular hydrogel had enhanced permeation as compared to plain hydrogel owing to the flexible and squeezable nature of vesicles.^84,85^

 Nanocarriers can avoid the passage via the epidermal layer by using hair follicles as passageways. The hair follicles can be deposited with lipid-based nanocarriers, which act as a storehouse for the drug’s prolonged constant release. Pereira et al developed rifampicin and clindamycin-loaded NLCs for the therapy of Hidradenitis suppurativa, a condition in which pilosebaceous units get permanently blocked. The findings demonstrated the accumulation of nanoparticles in hair follicle openings which act as depots for the drug’s sustained release.^86^ Via the follicular ducts, lipoidal nanocarriers can be transported to the skin’s underlying layers.^87^

## Recent lipoidal nanoformulations advancements for gout management

 Since lipid-based nanocarriers have such outstanding features as minimal toxicity, great biocompatibility, minimal cost of manufacturing scale-up, and high drug loading efficiency, they have drawn a lot of interest as carriers of pharmaceuticals with low oral bioavailability.^88^ The research advancements with different categories of lipid nanoparticles are given below:

###  Transferosomes

 One of the most effective therapies for gout is colchicine. It belongs to BCS Class-III and, thus possesses high solubility and poor permeability via skin/membrane. In addition, it also shows GI side effects like diarrhoea when given orally. Nevertheless, at therapeutic dosages, it is linked to adverse effects in eighty percent of users. Moreover, it is a strong alkali with a pKa of approximately 12.8 that has water solubility, ionizing at the physiological pH of the GIT tract, resulting in a limited oral bioavailability of 44%.^80^ El-Feky and colleagues developed transferosomes for transdermal delivery, improving Colchicine’s absorption and mitigating its adverse effects. To overcome the problem of drug leakage from transferosomes, colchicine was complexed with Beta-cyclodextrins before formulation. The developed colchicine-beta-cyclodextrin complex-loaded transferosomes were further evaluated for particle size, shape, percent entrapment, flexibility, and *in vitro* drug release. Furthermore, the formulations were also tested for permeation via skin, preclinical studies, and histopathology. The minimum particle size and the maximum entrapment found are 70.6 nm and 93.8% respectively. A biphasic controlled release pattern was observed with significant permeation potential, enhanced effectiveness, and decreased skin irritation.^89^

 The other active moiety for the treatment of gout, Allopurinol, a purine derivative xanthine oxidase inhibitor, is also a poorly soluble drug. Tiwari et al formulated transferosomes of allopurinol to enhance its penetration via skin and then loaded them in hydrogel formulations. Transferosomes were developed by the thin-film hydration technique using different ratios of tween 80 and soya lecithin. The developed formulations had controlled particle size with the highest entrapment of around 83%. The surface morphology was found to be spherical with a zeta potential of -29 mV, demonstrating excellent stability. The formulation revealed zero-order release kinetics with a cumulative release of approximately 81.7% at the end of 8h. On applying transferosomal gel, no sensitivity or irritation was found after seven days, while the allopurinol-loaded plain gel showed patchy erythema. This might be due to allopurinol’s high molecular weight, which resists its penetration via skin.^23^

###  Solid lipid nanoparticles

**Figure 4:**
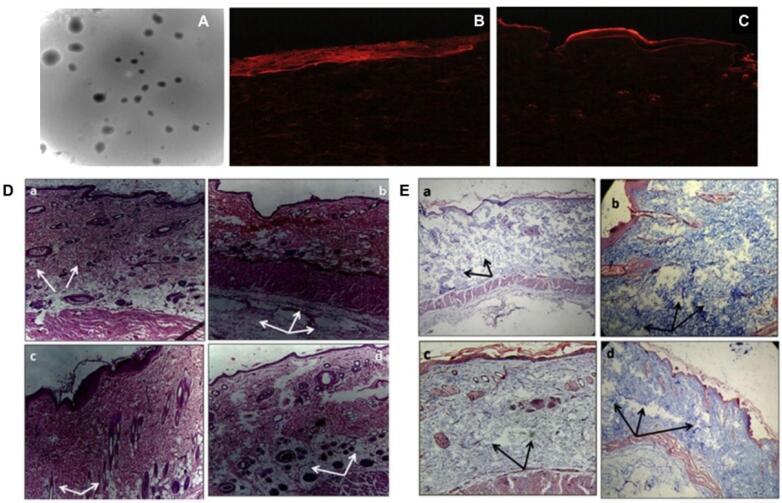
(A) TEM image of colchicine-SLNs; (B) Picture showing the deposition of rhodamine-B solution in rat skin; (C) Picture showing enhanced permeation and deposition of rhodamine-B loaded SLNs in rat skin; (D) and (E) Rat air-pouch model: rat skin stained with Eosin-hematoxylin (a) no-treatment group (b) PBS control group (c) Colchicine-SLN patch treated group (d) plain-colchicine patch treated group. (White arrows indicate inflammatory cell accumulation while black arrows show the deposition of collagen).Reprinted from Joshi et al^89^ Copyright (2025), with permission from Elsevier

 Another cohort developed SLNs of colchicine by ultrasonication technique using glyceryl monostearate as lipid, tween 80, and sodium lauryl sulphate as surfactant. Later, these nanoparticles are loaded on a transdermal patch for easy application (Figure 4). The size of the developed particle was found to be 107 nm with entrapment of around 37%. The transdermal gradient of developed SLNs was high, with greater increased potential for skin penetration as per fluorescence testing. The treatment with colchicine-loaded SLN transdermal patch resulted in a substantial (*P* < 0.001) decrease in WBC count, volume of exudate, and collagen accumulation in the Wistar rats by subcutaneous air pouch model.^27^

###  Nanostructure lipid carriers

 Unlike Allopurinol, Febuxostat, a non-purine xanthine oxidase inhibitor, belongs to the BCS class-II category with poor solubility and good permeability. To enhance its physicochemical properties, Sharma et al developed febuxostat’s NLCs by using stearic acid and oleic acid as solid and liquid lipids, respectively, with the help of the hot-pressure homogenization technique and subsequently loaded it in a hydrogel formulation for ease of application. The developed formulation demonstrated a release of 87% in six hours. Although the study lacks *ex vivo* permeation and preclinical studies, which could have given better insight into the role of NLCs for the delivery of febuxostat via the topical route for gout management.^26^

###  Ethosomes

 Furthermore, with the use of different concentrations of soya lecithin and ethanol, El-Shenawy et al, formulated ethosomes of febuxostat by cold method and loaded them in hydroxypropyl methyl cellulose-based gel. The developed formulations demonstrated a minimum particle size of 124.2 nm with PDI below 0.5. The zeta potential was found to be high i.e., -43.5 mV confirming the stability of ethosomal suspension. When ethosomes are compared to febuxostat-plain gel, the ex vivo permeation studies show a superior penetration profile. Based on the preclinical findings, transdermal delivery of febuxostat produced considerably greater Cmax and tmax values compared to when taken orally.^90^

###  Cubosomes

**Figure 5:**
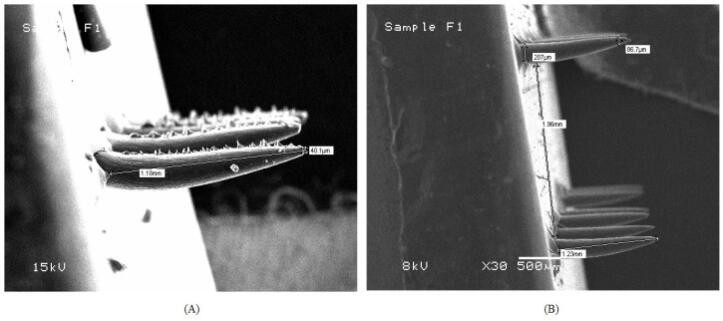
Scanning Electron microscopic images of (A) Febuxostat loaded microneedle patch; (B) Febuxostat cubosomes loaded microneedle patch. Reprinted under the terms and conditions of the Creative Commons Attribution (CC BY) license (https://creativecommons.org/licenses/by/4.0/).^91^

 A research group had formulated febuxostat cubosomes by bottom-up approach for its enhanced penetration via the topical route. Cubosomes are composed of lipids like glyceryl monooleate, surfactants like poloxamer 407 and polyvinyl alcohol, and water that results in the formation of cubic-liquid crystalline phase nanoparticles. The group then loaded developed cubosomes into microneedles using a micromolding approach. For the optimization of cubosomes and microneedles, the Quality-by-Development approach was used. Particle size and entrapment efficiency for cubosomes and axial fracture force and dissolving time for microneedles were the important quality attributes that were chosen. Figure 5 shows the SEM photomicrograph of Based on the ex vivo investigation, the cubosomes-loaded microneedle patch demonstrated the maximum transdermal flux, after FBX cubosomes. Based on the pharmacokinetic analysis, the cubosome-loaded MN patch was found to be the most effective way to penetrate the stratum corneum and transfer febuxostat to the blood via the transdermal route, outperforming the cubosomal gel. In the pharmacodynamic examination, the febuxostat-loaded cubosomal gel of FBX was found to be less effective in controlling the UA concentrations in rat blood, as compared to the febuxostat-loaded microneedles patch.^91^

###  Nanoemulsions

 Apart from NLCs, cubosomes, and ethosomes, nanoemulsions have also been found as promising formulations for the delivery of BCS class-II drugs via the topical route. Kanke et al developed febuxostat-loaded nanoemulsions using Capmul MCM as oil phase, tween 80 and transcutol P as surfactant and co-surfactant, respectively, with the help of an aqueous titration technique. The average globule size of the optimized formulation was found to be less than 55 nm with a PDI of 0.33 and a relative viscosity of 30.22 cps. Steady-state flux was found to be significantly enhanced in nanoemulsion formulation. The addition of more studies like preclinical would have given a good idea about the function of nanoemulsion in enhancing permeability.^92^

###  Self-nanoemulsifying drug delivery system 

 Alhakamy et al developed a SNEDDS for the solubility enhancement of febuxostat and loaded SNEDDS in a transdermal film for topical delivery. The group developed eight SNEDDS formulations with the use of lemon oil, tween-20, and polyethylene glycol-400 as surfactant and co-surfactant, respectively. The minimum globule size of SNEDDS was found to be 177 nm. The optimized formula demonstrated enhanced skin permeation, which was confirmed by fluorescence analysis. When compared to plain febuxostat film, the preclinical findings revealed a substantial (*P* < 0.05) change in pharmacokinetic characteristics and drug concentration in plasma.^93^

## Clinical trials on lipoidal nanoformulations for gout management

 Lipid-based carrier is explored as a novel carrier for anti-arthritic drugs, especially for anti-inflammatory and analgesic, due to prolonged release and improved therapeutic effect. Clinical implications are important parameters that can be used to confirm the translation of research to the community’s needs. Several preclinical studies have been reported, however, only a few reaches clinical trials which is evident from the figure count of liposomal products in the market. This may be attributed to insufficient data to conclude outcomes, or the product is not cost-effective. Table 2 summarises the different clinical trials done by using liposomal formulation in arthritis treatment. Most of the clinical trials were done on liposomal bupivacaine for postoperative pain management and compared with several conventional methods for their effectiveness and equivalency as alternative methods. The outcome of all completed clinical trials indicates the superiority of liposomal formulation compared to conventional methods and drugs in pain management and inflammation control. Most clinical trials were done by interventional studies either open-label or single-level blinded, only a few interventions were double-blinded. Details of a few clinical trials have been summarized in Table 2.

**Table 2:** Compiled list of lipoidal formulations under clinical trials for the treatment of gouty arthritis

**Clinical trial number and sponsor**	**Objective**	**Drug**	**Status and Phase**	**Study type**	**Reference**
NCT00241982 Radboud University Medical Centre	Safety evaluation of sustained released liposomes after IV administration for treatment of active rheumatoid arthritis inpatient	Prednisolone	Completed Phase II	Interventional (Parallel assignment) Double (Participant Care Provider)	^94^
NCT02276040 OrthoCarolina Research Institute, Inc.	This study was designed to evaluate the serum concentration of the drug in OrthoPAT® collected blood at definite time intervals. The purpose is to investigate the toxicity of the drug after reinfusion of blood from OrthoPAT® after total joint arthroplasty.	Bupivacaine	Completed	Observational- Case-Only Prospective	^95^
NCT03219983 The Christ Hospital	Clinical trial was done to do a comparative evaluation of pain management after total shoulder arthroplasty with local tissue infiltration of liposomal bupivacaine and Ropivacaine via interscalene block under ultrasound guidance.	Bupivacaine Ropivacaine	Terminated Phase IV	Interventional (Parallel assignment)	^96^
NCT02341079 United States Naval Medical Center, San Diego	The study was done to investigate the alternative method equivalency of infiltration of liposomal bupivacaine with indwelling femoral peripheral nerve block for postoperative pain management in knee arthroplasty.	Bupivacaine	Withdrawn Phase 2 Phase 3	Interventional (Parallel assignment) Open Label	^97^
NCT02787226 Eric Shepard, University of Maryland	To investigate the new method to prevent postoperative pain without a catheter to deliver ropivacaine directly to the shoulder nerve via using liposomal bupivacaine.	Bupivacaine Ropivacaine	Terminated Phase IV	Interventional (Parallel assignment) Open Label	^98^
NCT02166632 Broward Health	The investigation was done to know the difference in the delivery of liposomal bupivacaine in two different ways during surgery and 2^nd^ by injecting in periarticular tissue as local infiltration anesthetic.	Bupivacaine	Completed	Interventional (Parallel assignment) Single level masking	^99^
NCT02197273 OhioHealth	A Comparative interventional study for pain management between Liposomal Bupivacaine and standard drug for pain relief. arthroplasty, as compared to standard of care analgesia.	Bupivacaine	Completed	Interventional (Parallel assignment) Single level masking	^100^

## Patents in lipoidal nanocarriers-based arthritis therapy

 The patent shows the innovation and implementation of the new idea for technology protection. In the area of liposomes, several patents were filed and granted for arthritis treatment. One patent claimed that a liposomal formulation containing therapeutic agents can give longer symptomatic relief for arthritis and help to reduce the frequency of IA injections compared to the current treatment regimen (US9789062B2). Similarly, adenosine-encapsulated liposomes of sphingomyelin and its derivative were developed to induce cartilage regeneration, pain management, and alleviate tissue damage. They claimed that adenosine would be released for 2 weeks, which would reduce the dosing frequency (WO2020206314A1). A cationic liposome was prepared and loaded with polypeptide active molecules to treat autoimmune diseases and inflammatory disorders such as rheumatoid arthritis (WO2007134819A1). Several other patents on different types of arthritis are summarised in Table 3. It is evident from Table 3 that most patents are focused on RA treatment, especially for treating inflammation. Most patents are meant for the local delivery of anti-inflammatory drugs or pain management in various arthritis.

**Table 3:** List of lipoidal formulation-based patents published/granted for gouty arthritis

**Application number**	**Patent Claim**	**Patent status/ Year**	**Reference**
US9789062B2	Liposomal formulation with a therapeutic agent reduces IA injection frequency and produces long-term effects.	Application granted 2017	^101^
WO2020206314A1	Sustained release of Adenosine-loaded liposomes to improve pain and tissue damage.	Published 2021	^102^
WO2007134819A1	Development of cationic liposomes for targeted delivery of polypeptide and other API.	Published 2007	^102^
WO 01/82899	Development of cationic liposome for diagnosis and delivery of therapeutic drug at the active site to treat inflammation in arthritis.	Published 2003	^103^
EP 2638896 A1	Targeting of human cd14 + monocytes in blood by cationic liposome for RA treatment.	Published 2013	^104^
EP 1658839 A1	Cationic liposomes loaded with Oligonucleotides exert site-specific anti-inflammatory effects in RA by targeting CD40	Published 2006	^105^
WO 2003000190 A2	Intraarticular delivery of glycosaminoglycan encapsulated liposomes for osteoarthritis treatment	Published 2003	^106^
WO 2006027786 A2	Liposomal preparation containing glucocorticoid, and its derivative was developed to treat inflammatory conditions of rheumatoid arthritis.	Published 2006	^107^
WO 2008053484 A2	Development of targeted nano liposome with heavy metal to treat inflammation in RA.	Published 2009	^108^
US8784881B2	Curcuminoid-loaded liposomes for anti-inflammatory effect on arthritis	Published 2014	^109^
US 20130115270 A1	Targeted liposomes attached with Anti-IL-1 for local anti-inflammatory action in RA treatment	Published 2013	^110^
US 20130071321 A1	Targeted liposome with folate conjugation for site-specific delivery of corticosteroid.	Published 2013	^111^
US 20130115269 A1	NF-a coated liposomes for targeted delivery of anti-inflammatory drugs	Published 2013	^112^

###  Challenges in clinical applications

 Lipid-based nanocarriers, while promising for drug delivery and therapeutic applications, face challenges in economic scalability, regulatory hurdles, and clinical limitations that hinder their widespread adoption. Lipid nanoparticles are prone to fusion, especially when generated nanoformulations are less than 100 nm in size. The fusion allows the enclosed contents to escape from the lipid vesicles, improving dispersity. NLCs made with surfactants like poloxamer 188 and polysorbate 80 showed low toxicity and adequate biocompatibility. It has also been observed that using a surfactant combination to improve product stability may raise the risk of toxicity. Numerous studies have shown that lipid-based nanocarriers produced using positively charged cationic surfactants such as cetyltrimethylammonium bromide have adequate cellular tolerability.^107^

 Stability is an important need in the commercial manufacturing of LNP formulations, and it can be modified by lipid polymorphism or phase changes. During preparation or storage, triglycerides in LNPs may change from α- to β-form, resulting in polymorphic crystalline aggregates and decreased amorphous zones in the carrier matrix. This can lead to drug leakage. Sterilizing LNPs during industrial manufacturing is difficult due to the risk of instability produced by current procedures. For example, γ radiation, often used for sterilizing, can cause lipid oxidation and chain scission, decreasing LNP stability and effectiveness. Furthermore, lipid oxidation during storage might affect particle surface charge, drug release characteristics, and stability, potentially leading to the generation of hazardous byproducts that impair therapeutic efficacy. Furthermore, interactions with container materials such as ion leaching, surfactant absorption, and pH changes can all have an impact on LNP stability. Because of these difficulties, most LNP formulations have a shelf life of less than a year. To address these issues, a variety of strategies have been developed, including lyophilization, the use of stabilizing agents such as antioxidants or chelators to prevent oxidation and aggregation, the use of excipients as buffers, osmolytes, or cryoprotectants, and specialized packaging materials to prevent container interaction.^108^

 In addition to the obstacles of research and development, the regulatory clearance procedure is expected to be a further hurdle for potential LNP-based solutions, especially given the wide spectrum of chemicals that LNPs may provide. To guarantee regulatory compliance, toxicological tests are critical, especially for LNPs, because their accumulation in healthy tissues can cause cytotoxicity and genotoxicity. This might be because of the cationic lipid components included in LNP formulations. Stearyl amine, a first-generation monoalkyl cationic lipid, has been found to produce erythrocyte hemolysis and hemagglutination. LNP-based medications are subjected to rigorous testing throughout clinical trial stages 1-3, which include evaluations of healthy persons, patients with the target condition, and the public. To make educated judgments about allowing a medicine for commercial use, the regulatory agency evaluates its safety, efficacy, probable adverse effects, dosage regimen, and overall safety profile.^108,109^

## Conclusion

 Gout is a condition that is highly disregarded and inadequately treated. Conventional treatment approaches have only been able to lessen the condition’s severity by symptom relief. Acute GA typically has an immediate, aggressive onset; however, the actives frequently must circulate within the blood before they arrive at the target site, which slows down their rate of action and increases the risk of profound side effects. Moreover, most gout-relieving medications possess inadequate targeting, short half-lives, and insufficient solubility in water, which leads to inadequate bioavailability and systemic toxicity. Enhancing the safety, effectiveness, and bioavailability of the available medications is the main goal of innovative formulation approaches like nanotechnology-based drug delivery.

 The topical application of lipid-based nanocarriers for GA is a potentially significant development in treating this long-term ailment. Lipoidal nanocarriers provide several benefits, such as increased penetration through the skin, better drug solubility, controlled drug delivery and site-specific delivery to arthritic joints which may lower application frequency and increase therapy adherence. Such characteristics minimize systemic adverse effects while facilitating the attainment of significant local drug levels at the site of inflammatory processes. Furthermore, lipid nanocarriers are a safe and efficient approach for prolonged drug release owing to their biological compatibility and biodegradable properties.

 Lipid-based nanocarriers could have exceptionally promising prospects in gout therapy, featuring potential developments that could greatly improve the effectiveness of treatment. Subsequent investigations could concentrate on creating multifunctional lipid nanocarriers that incorporate urate-lowering and anti-inflammatory actives in a unit-dose formulation. This might offer a thorough approach to treating gout by addressing both its symptoms and their root cause. Biologics or gene therapy may be delivered using lipid nanocarriers, which may provide a long-term remedy or a curative treatment for the gout condition. This strategy has the potential to completely change the way that severe situations of gout are treated. With the ability to replace or supplement oral drugs, lipid nanocarriers can provide patient-friendly, non-invasive therapy choices including topical formulations that are simple to administer, hence increasing patient adherence and quality of life. Enhancements in stimulus-responsive LNPs, which deliver medicine concerning stimuli such as pH shifts in inflammatory tissues, may result in a more efficient and targeted treatment for gout.

## Competing Interests

 The authors declare no conflict of interest.

## Consent for Publication

 The authors declare no conflict of interest.

## Data Availability Statement

 Data sharing not applicable to this article as no datasets were generated or analysed during the current study.

## Ethical Approval

 Not applicable.
